# Comparison of executive functions in individuals with high and low levels of schizotypal trait

**DOI:** 10.3389/fpsyg.2022.1071777

**Published:** 2023-01-27

**Authors:** Saeid Abbasi Sarajehlou, Somayeh Khajeh, Cheeman Masrour, Morteza Azizi

**Affiliations:** ^1^Department of Psychology, Faculty of Education and Psychology, University of Tabriz, Tabriz, Iran; ^2^Department of Psychology, Science and Research Branch, Islamic Azad University, Tehran, Iran; ^3^Department of Psychology, Sarab Branch, Islamic Azad University, Sarab, Iran

**Keywords:** executive functions, personality disorders, schizotypal traits, schizotypal personality disorder, personality, personality traits

## Abstract

The dimensional approach to Schizotypal Personality Disorder (SPD) indicates that SPD includes a constellation of maladaptive personality traits on a continuum with general personality functioning. This study aimed to compare executive functions (EFs) in low and high-level schizotypal individuals. Using a convenient sampling method, we recruited 120 individuals, from which 30 individuals with high schizotypal trait levels (fourth quartile) and 30 participants with lower schizotypal trait levels (first quartile) were selected based on their scores on the schizotypal personality disorder questionnaire. Then, participants from the two groups were administered the Corsi Block-Tapping Test (CBTT), Wisconsin Card Sorting Test (WCST), and Continuance Performance Test (CPT). The results indicated individuals with higher schizotypy trait levels performed significantly poorer in tasks measuring working-visual–spatial memory, cognitive flexibility, sustained attention, and response inhibition. This pattern of results indicated that EF dysfunctions in individuals with higher schizotypy trait levels would cause significant disturbances in multiple areas of life. The practical implications of the findings are further discussed.

## Introduction

According to the fifth definition of the Diagnostic and Statistical Manual of Mental Disorders (DSM-5), Schizotypal Personality Disorder (STPD) is a “pervasive pattern of social and interpersonal deficits marked by acute discomfort with, and reduced capacity for, close relationships as well as by cognitive or perceptual distortions and eccentricities of behavior, beginning by early adulthood and present in a variety of contexts.” STPD is diagnosed when individuals meet at least five of the nine diagnostic criteria (American Psychiatric Association, 2013). However, many studies have demonstrated the shortcomings of the current DSM diagnostic system, including inadequate scientific basis, arbitrary cutoffs, comorbidity among personality disorders (PDs), comorbidity with other psychological disorders, heterogeneity of diagnoses, and insufficient coverage. Also, concern has been raised with respect to all the personality disorders within DSM-IV-TR, including the STPD, on whether they are best understood as qualitatively distinct syndromes or constellations of maladaptive personality traits that are on a continuum with general personality functioning. These shortcomings have led to the dimensional approach to PDs (e.g., Edens et al., 2008; Arntz et al., 2009; Samuel et al., 2013; Southward and Cheavens, 2018; Elhami Athar et al., 2022). This approach is reflected in the alternative model for the diagnosis of personality disorders (PD), published in DSM-5’s section III, i.e., emerging models and measures that need further research (American Psychiatric Association, 2013). In this vein, studies indicate that STPD has a dimensional structure and includes components that reflect different levels of pathology (Edmundson et al., 2011; Kemp et al., 2022). Consistent with this approach, from the perspective of the five-factor model of general personality (FFM; McCrae and Costa, 2003), Whiteside and Lynam (2001) developed the Five-Factor Schizotypal Inventory (FFSI), which includes nine schizotypy scales as maladaptive variants of respective facets of the FFM (e.g., Aberrant Ideas as a maladaptive variant of FFM Openness to Ideas).

Furthermore, there is extensive evidence that STPD is part of the schizophrenia spectrum, with both genetic links and similarities in biological and psychological correlates (e.g., Schultze-Lutter et al., 2019; Pries et al., 2020). Consistent with these findings, DSM-5 has included a new category, namely Schizophrenia Spectrum and Other Psychotic Disorders, which includes psychosis-related disorders along with the STPD (which also falls under the category of personality disorders). While many etiological factors have been suggested for the development of schizophrenia spectrum disorders (SSP), a predominant hypothesis indicates that frontal lobe dysfunction characterizes SSP, including STPD (e.g., Green et al., 2000; Ettinger et al., 2015; Medalia et al., 2018). Deficits in the frontal lobe are associated with disturbances in Executive Functions (EFs)—a set of general-purpose controlling mechanisms that control the dynamics of human actions and cognition (Goldstein and Naglieri, 2014). EFs allow one to receive environmental stimuli, respond adaptably, change their path, predict future purposes, consider consequences, and respond consistently and logically. The core components of EF are cognitive flexibility/shifting, response inhibition, and working memory (Goldstein and Naglieri, 2014). People with high degrees of schizotypy features have been found to have worse neurocognitive performance, albeit in a milder form, comparable to that seen in schizophrenia (Gooding et al., 1999; Matheson and Langdon, 2008; Meller et al., 2019; Gilleen et al., 2020). In a review of cognition in schizotypy, Ettinger et al. (2015) identified several perceptual, cognitive, and motor functions that deteriorate in individuals with higher schizotypy. The authors concluded that while there is generally consistent evidence of impairments in certain perceptual-motor abnormalities, there is considerable cross-study variability in higher cognitive deficits such as working memory or top-down attentional control. Ettinger et al. (2015) suggested future research identify areas of impairment in high and low schizotypy and characterize areas of function that may be spared in high schizotypy compared to schizophrenia. Accordingly, such studies will increase knowledge of the cognitive processing pattern in individuals with high schizotypy and will also inform and contribute to the etiological theories of schizophrenia. Research has also revealed relative deficits in verbal and spatial working memory among individuals with schizotypy features. For instance, Matheson and Langdon (2008) found that once age was controlled for verbal working memory, as evidenced by correct manipulations on the Letter–Number Sequencing Task, it was a significant predictor of cognitive, perceptual, and negative interpersonal schizotypal traits. Additionally, mild forms of cognitive deficits seen in schizophrenia can also be found in unaffected first-degree relatives and healthy people with schizotypal traits (Cadenhead and Braff, 2002; Zhang et al., 2018; Jing et al., 2019). Taken together, these findings suggest that schizotypy traits are associated with lower cognitive ability.

To the best of our knowledge, overall, few studies have examined the associations between schizotypal traits and deficits in EFs. Furthermore, some past studies relied on data from clinical and adolescent samples, which restricts the generalizability of the findings to other samples. Examining schizotypal traits in non-clinical samples allows one to study neuropsychological impairment without potentially confounding factors such as medication, hospitalization, and developmental course. In addition, studying schizotypal traits in non-clinical samples provides information on the range of symptoms and associated neuropsychological impairments in the population in terms of their presence in individuals who do not seek treatment (Gur, 2016). Therefore, in the current study, we aim to compare EFs in a sample of university students with high and low levels of schizotypal trait levels.

## Materials and methods

### Participants and procedure

We first recruited 120 (aged 19–26 years, M_age_ = 23.24, SD = 4.89, 46.66% females) students from Tabriz University who completed the Schizotypal Personality Questionnaire (SPQ). Then, we selected 30 (M_age_ = 22.56, SD = 4.24, 46.66% women) individuals with higher schizotypal trait levels (the fourth quartile) and 30 (M_age_ = 23.02, SD = 4.42, 46.66% women) participants with schizotypal trait levels (the first quartile) who were administered the executive functioning tests. The inclusion criteria consisted of having the interest to participate in the study, while the exclusion criteria consisted of having a history of psychiatric disorder and brain surgery, which was assessed through the self-report information. The present study was first evaluated by the ethical committee of the University of Tabriz. Prior to gathering the data, participants provided a signed informed consent after being informed about the aims and process of the study and the confidentiality of the data.

### Measures

#### Schizotypal personality questionnaire

The SPQ is a self-report measure developed by (Raine, 1991) for evaluating the total spectrum of schizotypal symptoms, including nine features of schizotypal personality disorder based on DSM-III criteria. SPQ includes 74 items rated based on a dichotomous response format (Yes/No), which load on three subscales of cognitive-perceptual deficits, interpersonal deficits, and disorganization, with higher scores indicating greater schizotypy levels. The original study indicated that the SPQ enjoys adequate psychometric properties in terms of internal consistency and validity. Bayrami et al. (2011) supported the originally proposed three-factor structure for the Persian version of SPQ and showed that the Persian SPQ has adequate internal consistency and validity.

### Executive functioning tests

#### Continuance performance test

The Continuance Performance Test (CPT; Strauss et al., 2006) is a gold-standard clinical test of attention and response inhibition and is administrable for the age range of 6–55 years old. In this task, the English alphabet letters of A, B, C, D, E, F, G, H, I, J, L, M, O, P, Q, R, S, U, and X are randomly presented, one at a time, in the center of the computer screen, with all letters except ‘X’ being the target for the response. Participants are instructed to respond by pressing the spacebar of a computer when any target letters appear. Letters appeared at intervals of 1,000, 2,000, and 4,000 ms. Twenty stimuli were created by dividing 360 stimuli into 18 blocks. Each stimulus was randomly presented three times with intervals between each presentation. The results yield three variables: omission errors (for evaluating the status of sustained attention), commission errors (for evaluating inhibition), and response time. The overall administration time takes 14 min.

#### Wisconsin card sorting test

Wisconsin card sorting test (WCST) was developed by Grant and Berg (1948) to evaluate abstract reasoning ability and the ability to shift cognitive strategies. We used the version with 64 cards. On each of the cards, the triangular, star, cross, and circle signs of 1–4 have been printed in red, green, yellow, and blue colors; none of the cards are similar to each other. The quizzer puts 4 sample cards in front of the subject, including one red triangle, two green stars, three yellow crosses, and four blue circles, and asks him to put the rest of the cards one by one below the four primary cards (according to the feature of each sample card). This is done based on a principle that the subject has to conclude based on correct and wrong responses of the quizzer over putting the cards. Shahgholian et al. (2011) supported the psychometrics of the computerized version of Persian WCST.

#### Corsi block-tapping test

Corsi Block-Tapping Test (CBTT) is one of the most practical tools for assessing visuospatial short-term memory (Berch et al., 1998). This test was designed based on the digital span test, but instead of the verbal form existing in the digital span test, this test requires working visual–spatial memory. Corsi test’s process is such that the subject sees nine blocks on the computer screen and some of these blocks turn on with a specific sequence in each effort. The subject’s task is to remember the sequence of the blocks turning on and click on the blocks to repeat the series after the process is finished. This test starts with two blocks, but the number of blocks increases with each effort. The task lasts for nine blocks, but in case of two errors in one sequence, the test finishes, and the longest remembered sequence of the subject is recorded. Overall, the mean score for ordinary people is five blocks. Walker et al. (2010) obtained a test–retest reliability of 0.73 for CBTT. The Persian version of the CBBT yielded reliable results in previous studies with Iranian samples (e.g., Elhami Athar et al., 2020).

### Data analyses

We used SPSS 22 software for data entry and statistical analyses. First, we conducted Kolmogorov–Smirnov test to examine the normality of the distribution for the study variables. Then, a series of independent t-test were used to compare the two groups in the EFs scores. It was decided beforehand that a *p* level of less than 0.05 would be accepted as indicating statistically significant results.

## Results

First, the normality of the distribution for the study variables was supported according to the Kolmogorov–Smirnov test results (0.05 < *p*). Next, we conducted a series of independent *t*-tests to examine the differences in working-visual–spatial memory, cognitive flexibility, response inhibition, sustained attention, and reaction time across the groups. As shown in Table 1, the results of the independent t-tests indicated that the group with low schizotypal traits perform significantly better than the high schizotypal group in the task related to working-visual–spatial memory [*t*(58) = 6.65, *p* = 0.001], cognitive flexibility, including perseveration errors [*t*(58) = −3.61, *p* = 0.001] and categories completed [*p* = 0.001; *t*(58) = 5.58, *p* = 0.001], response inhibition [*t*(58) = −5.64, *p* = 0.001], sustained attention [*t*(58) = −2.59, *p* = 0.001], and reaction time [*t*(58) = −4.06, *p* = 0.001].

**Table 1:** Descriptive statistics and comparison of the groups based EFs variables.

Task variable		Group	Mean (SD)	t	*p*	d
Corsi Block Tapping Test	Visuospatial Working Memory	Low	53.54 (14.21)	6.65	0.001	1.78
		High	27.43 (14.98)			
Wisconsin Card Sorting Test	Perseveration Error	Low	2.65 (2.51)	−3.61	0.001	0.97
		High	5.17 (2.66)			
	Categories Completed	Low	5.31 (1.12)	5.58	0.001	1.49
		High	3.33 (1.47)			
Continuance Performance Test	Commission Error	Low	0.58 (0.64)	−5.64	0.001	1.51
		High	3.80 (2.84)			
	Reaction Time	Low	1274.73 (85.99)	−0.2.59	0.01	0.69
		High	1336.27 (90.62)			
	Omission Error	Low	0.42 (0.76)			
	High	2.40 (2.37)	−0.4.06	0.001	1.09

SD, standard deviation; d, Cohen’s d.

## Discussion

This study aimed to compare executive functions in individuals with low and high schizotypal trait levels in a sample of university students. While most of the prior studies have examined executive functions in individuals diagnosed with schizotypal personality disorder, we considered schizotypy as constellations of maladaptive personality traits that are on a continuum with general personality functioning (e.g., Edens et al., 2008; Arntz et al., 2009; Samuel et al., 2013; Southward and Cheavens, 2018; Elhami Athar et al., 2022) and examined whether individuals with higher schizotypal traits levels perform poorer in executive functioning tests than those with lower levels of schizotypal traits. This type of study is also in line with suggestion of Ettinger et al. (2015) that studies on samples of individuals with high schizotypy will contribute to the etiological theories of schizophrenia.

Our results showed that individuals with lower schizotypal trait levels performed significantly better in the working-visual–spatial memory task (measured using the Corsi Block Tapping Test) than participants with higher schizotypal traits (Forbes et al., 2009; Schmidt-Hansen and Honey, 2009). Impairments in working memory are one of the critical features of schizophrenia, but more subtle impairments in working memory have been reported in relation to elevated levels of positive and negative schizotypy which may also explain some of the problems in the daily functioning of these individuals (For a review, see Ettinger et al., 2015). Our finding is consistent with the finding of Gooding and Tallent (2003) in that defects in working memory are mostly observed in schizophrenia-related disorders. Gooding and Tallent (2003) further indicated that individuals with higher schizotypal features have a lower ability to keep working memory representations. Likewise, Xie et al. (2018) indicated that individuals with schizotypal features tend to have less precise representations retained in visual working memory.

In the same vein, participants with lower schizotypal trait levels scored significantly better than their counterparts with high schizotypal trait levels in terms of cognitive flexibility as measured by WSCT, which is in line with the results of previous studies (Trestman et al., 1995; Diforio et al., 2000; Kwapil et al., 2008). These studies showed that frontal lobe dysfunctions in individuals with the diagnosis of schizotypal personality disorder might explain why they performed poorer in WSCT. The abilities to conceptualization, mental abstraction, and flexible thinking are parts of complex cognitive processes that are related to the prefrontal cortex, and all these abilities determine the performance in WSCT (e.g., Elhami Athar et al., 2020). Therefore, the reduction in the prefrontal cortex activity in individuals with higher schizotypal trait levels could reduce their performance in tasks demanding complex cognitive processes (Thomas et al., 2003).

Furthermore, our findings demonstrated that participants with higher schizotypal trait levels had significantly poorer response inhibition ability (measured by CPT) than those with lower schizotypal trait levels. The ability to inhibit responses to unrelated stimuli is one of the cognitive control criteria dependent on the proper functioning of the frontal lobe (Reddy et al., 2018). Our finding dovetails with the prior finding that compared to healthy people, those with schizotypal features have significantly disturbed function in tasks assessing response inhibition and responding time (Steel et al., 2007). Indeed, as explained above, the functional deficits in the frontal lobe of individuals with higher schizotypal trait levels could decrease their abilities in response inhibition. In this vein, Jia et al. (2021) used electroencephalography (EEG) to record the brain activity of participants while they were performing a task assessing response control. The results showed that individuals with schizotypy were impaired in both proactive and reactive response inhibition at behavioral and neural levels. Studies also show that individuals with high schizotypy perform poor in tasks assessing latent inhibition, i.e., a task in which exposure to an irrelevant stimulus prevents conditioning with the stimulus being formed at a later time (Kaplan and Lubow, 2011).

Finally, our results showed that participants with higher schizotypal trait levels had significantly poorer sustained attention ability (measured by CPT) than those with lower schizotypal trait levels. In support of our finding, Louise et al. (2015) indicated that higher scores of uncommon experiences, cognitive disorganization, and non-conformity of impulsivity were related to worse performance in the sustained attention task. Similarly, Trotman et al. (2006) showed that adolescents with the diagnosis of schizotypal personality disorder scored significantly lower scores than the normal control group in terms of the calculative sub-set of the Wechsler intelligence scale, which demands sound sustained attention. Selective attention to a given stimulus requires simultaneous focusing awareness on the target and inhibiting internal representations of the competing distractor. The inhibitory failures are identified as a core feature of schizophrenia since Bleuler. Individuals with schizophrenia and those with high levels of schizotypy show sustained attention deficits (Ettinger et al., 2015).

Overall, our results showed that individuals with higher schizotypal trait levels had significantly poorer performance in tasks measuring cognitive flexibility, working-visual–spatial memory, sustained attention, and response inhibition. EF dysfunctions in individuals with higher schizotypal levels would cause serious disturbances in their functioning in multiple areas. Therefore, it is necessary to consider suitable neuro-cognitive interventions for these individuals.

Our results should be interpreted considering a few limitations. First, we did not perform separate analyses for male and female samples. It is suggested that future studies examine if the disturbing effects of schizotypal trait levels on executive functions are similar across gender groups. Second, our results do not indicate the cause-and-effect relationship between schizotypal trait levels and performances in executive functions. Third, our study had a low sample size, so future studies could replicate our results with relatively larger samples. Finally, it would contribute to the literature if longitudinal studies explore the relationships between schizotypal features and brain structures using brain imaging.

## Data availability statement

The original contributions presented in the study are included in the article/supplementary material; further inquiries can be directed to the corresponding author.

## Ethics statement

The studies involving human participants were reviewed and approved by university of Tabriz. The patients/participants provided their written informed consent to participate in this study.

## Author contributions

SA gathered the data, performed the data analysis, and prepared the manuscript. SK and CM gathered the data. MA reviewed and revised the manuscript. All authors contributed to the article and approved the submitted version.

## Conflict of interest

The authors declare that the research was conducted in the absence of any commercial or financial relationships that could be construed as a potential conflict of interest.

## Publisher’s note

All claims expressed in this article are solely those of the authors and do not necessarily represent those of their affiliated organizations, or those of the publisher, the editors and the reviewers. Any product that may be evaluated in this article, or claim that may be made by its manufacturer, is not guaranteed or endorsed by the publisher.
